# Supplementary material for: KIAA0319 influences cilia length, cell migration and mechanical cell–substrate interaction
Source: Sci Rep. 2022 Jan 14;12:722. doi: 10.1038/s41598-021-04539-3 (PMC8760330; doi:10.1038/s41598-021-04539-3)
Supplement: Supplementary file 1 — Supplementary Information 1. [file 41598_2021_4539_MOESM1_ESM.pdf]

KIAA0319 influences cilia length, cell migration and mechanical cell-substrate interaction

Rebeca Diaz,[1]† Nils M. Kronenberg,[2,3]† Angela Martinelli,[1] Philipp Liehm,[2] Andrew C. Riches,[1] Malte C. Gather,[2,3]* Silvia Paracchini[1]*

† These authors have contributed equally to this paper

[1] School of Medicine, University of St Andrews, St Andrews, KY16 9TF, UK

[2] SUPA, School of Physics and Astronomy, University of St Andrews, St Andrews, KY16 9SS, UK

[3] Humboldt Centre for Nano- and Biophotonics, Department of Chemistry, University of Cologne, 50939 Cologne, Germany

* Corresponding authors:
Silvia Paracchini sp58@st-andrews.ac.uk
Malte Gather mcg6@st-andrews.ac.uk

Supplementary Figures

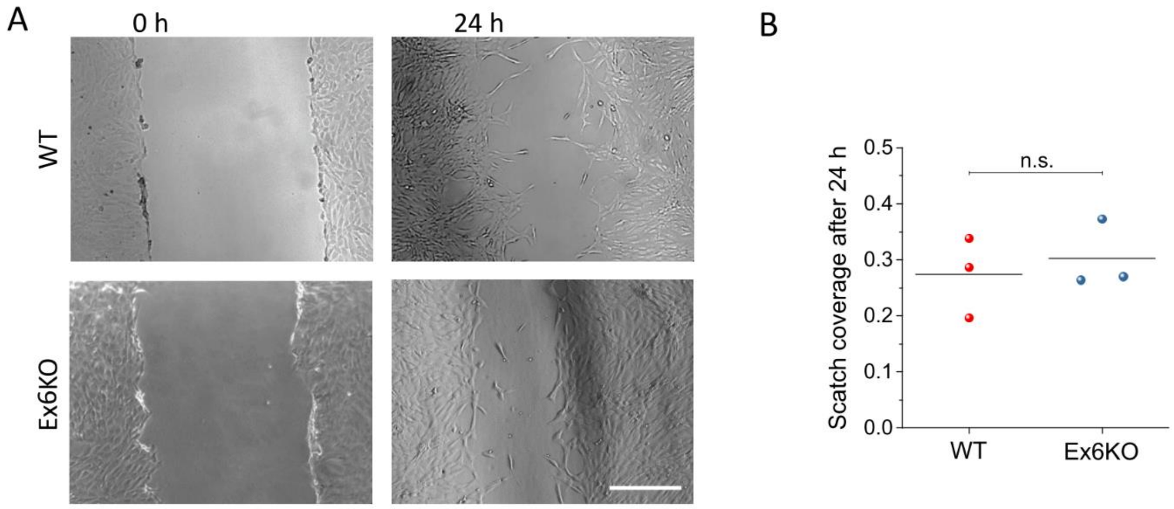

Figure S1. Scratch assay

(A) Representative phase contrast microscopy images of scratch assay at time 0 and after 24 h, for RPE wild type and Ex6KO. **(B)** Ratio of the scratch covered for each cell line after 24 h. Each dot represents an independent experiment ($n = 3$). There is no significant difference between the cell lines (*t*-test, $p = 0.63$). Scale bar, 500 µm.

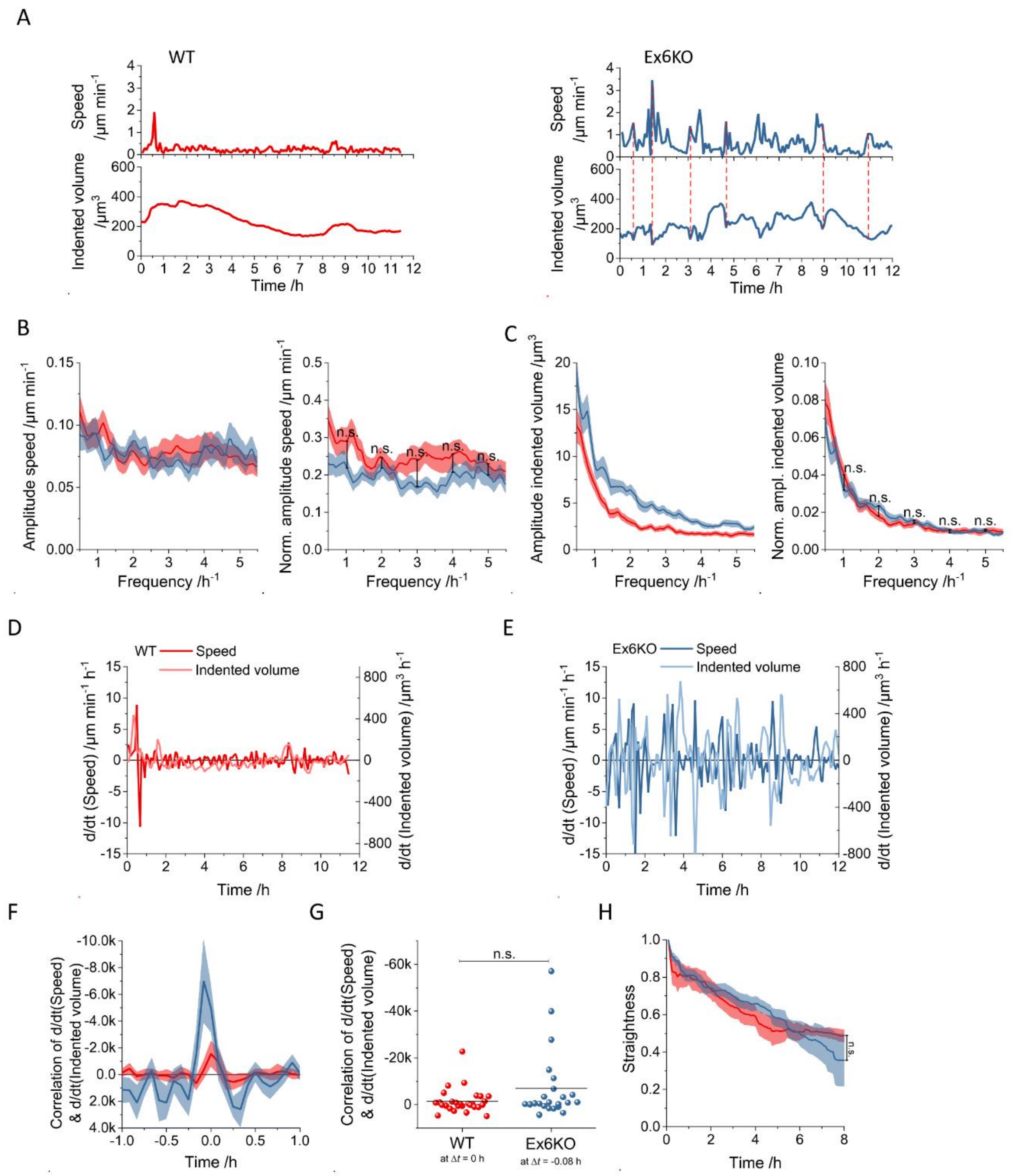

Figure S2. Analysis of mechanical activity of RPE1 WT and Ex6KO cells during migration.

(A) Exemplary temporal evolution of speed and mechanical activity (using the total volume by which each cell indents into the ERISM substrate as a proxy for the applied force) of representative RPE1 WT (left panel) and Ex6KO (right panel) cells, following the movement of two individual cells on an ERISM micro-cavity for >11 h. Red, vertical lines indicate time points when high migration speed of the Ex6KO cell correlate with a drop in exerted force. FFT analysis of amplitude of the oscillation of migration speed **(B)** and mechanical activity **(C)** (on left; data normalised to the mean speed and mechanical activity on right) of single RPE1 WT (red) and Ex6KO (blue) cells during migration on ERISM micro-

cavity. For significance testing via t-test, the amplitudes of the individual cells were compared separately in 1 h^{-1} frequency steps. WT: *n* = 29, Ex6KO: *n* = 24. **(D)** First time derivatives of speed and mechanical activity of the WT cell (left) and the Ex6KO cell (right) shown in Figure (A). **(F)** Mean temporal correlation between the first time derivatives of speed and mechanical activity for WT cells (red trace; *n* = 29) and Ex6KO cells (blue trace; *n* = 24). **(G)** Comparison of the peaks in temporal correlation between the first time derivatives of speed and mechanical activity for WT (*n* = 29) and Ex6KO (*n* = 24) cells. Each data point represents the measurement for one cell. The lines depict the positions of the means. Groups were compared using the Student's *t*-test (n.s.: $p > 0.05$). **(H)** Temporal evolution of straightness of WT (red) and Ex6KO (blue) single cell movement on ERISM micro-cavity. WT: *n* = 10, Ex6KO: *n* = 13. *t*-test was performed on data distributions after 8 h. In the plots in B, C, F and H lines depict the means and the shaded areas the standard error of the mean. Only cells with free movement for ≥2 h were included in the analysis.

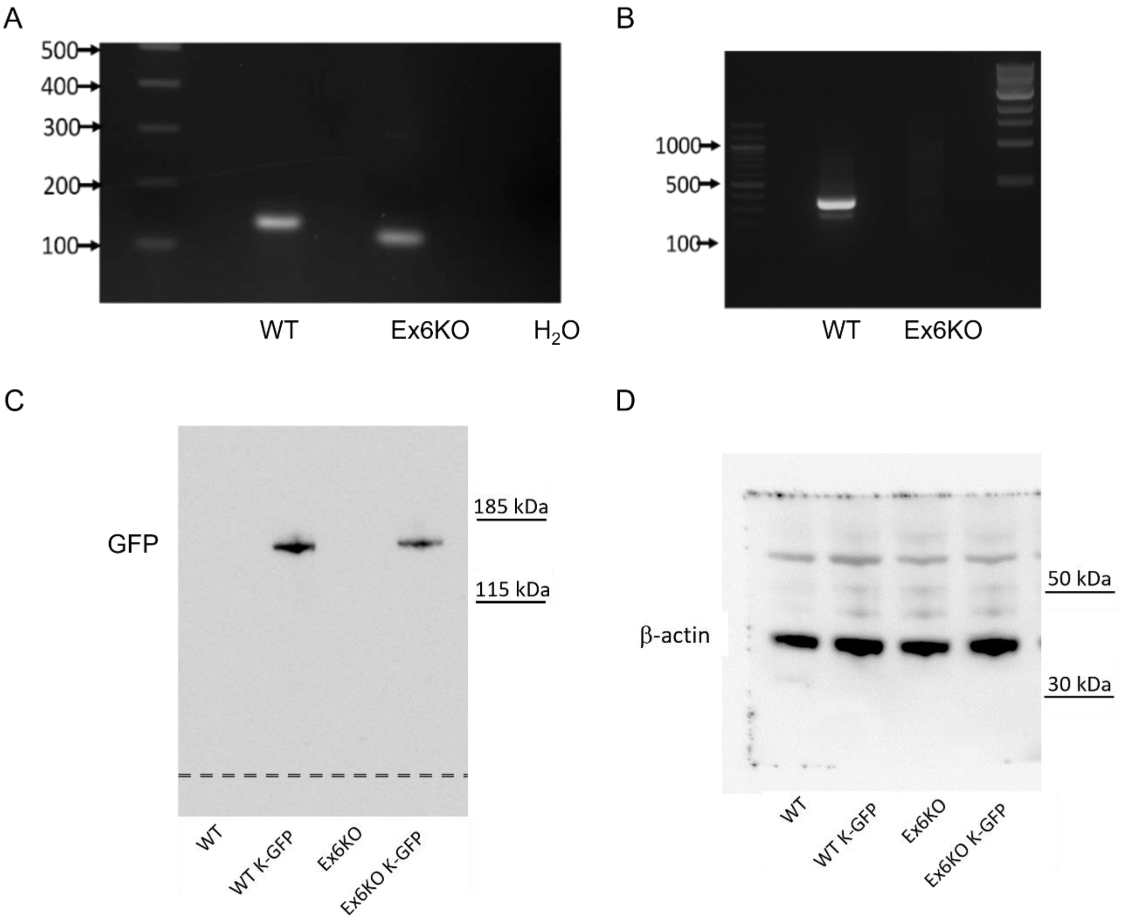

Figure S3. Uncropped images used in the manuscript. (A) and **(B)** Full-length gels for the cropped images shown in Figure 1C confirming the deletions in the exon 6 of KIAA0319. (**C)** Full length blot for the cropped image showed in Figure 4A confirming the presence of a fusion GFP protein following transfection with a full length KIAA0319 construct fused to a GFP tag (140 kDa). Due to the intensity of the signal, minimum exposure time was used to avoid overexposing the bands. Lower border of the membrane used is indicated with a dashed line. **(D)** Full length blot for the cropped image showed in Figure 4A for the corresponding β-actin loading control (40 kDa).

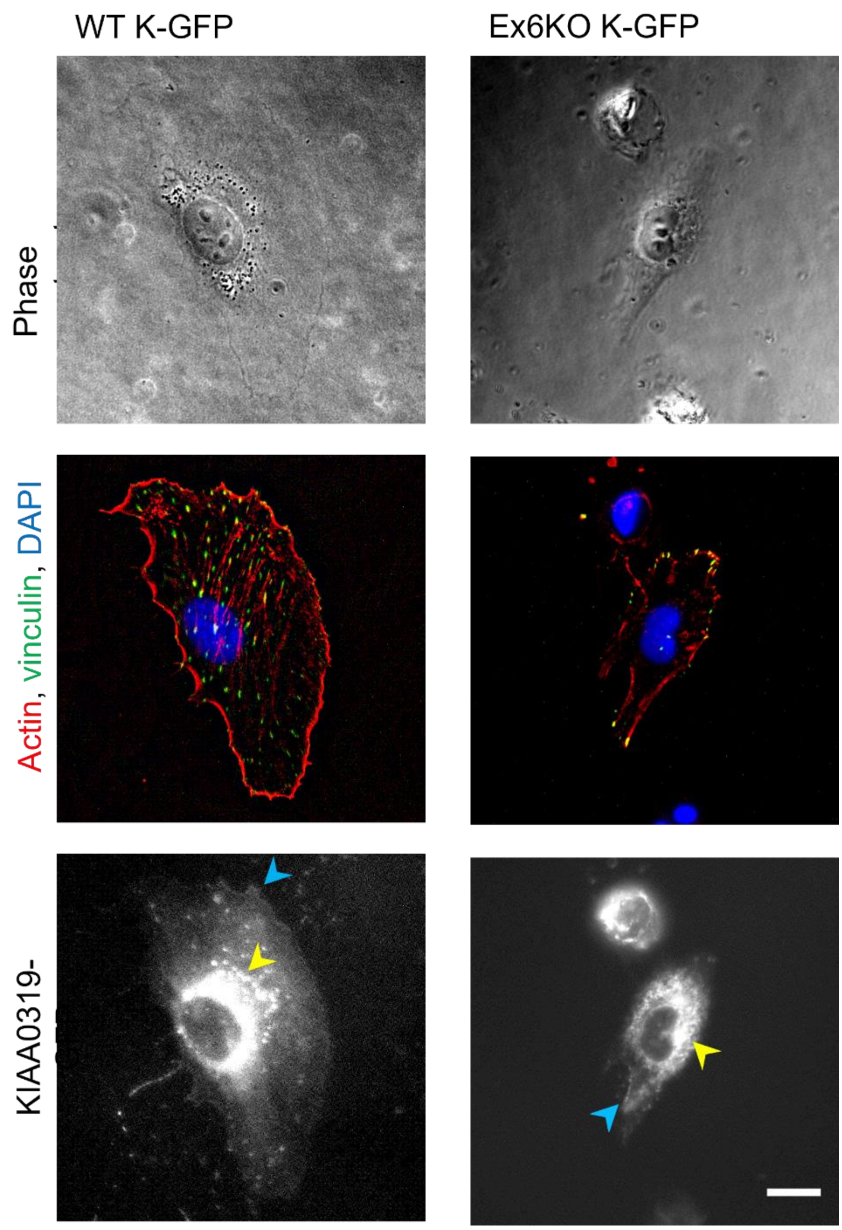

Figure S4. Phase contrast images (upper row) and epi-fluorescence images of actin (TRITC-conjugated phalloidin, red), vinculin (secondary antibody conjugated to Alexa Fluor 647, false-color green) and nuclear DNA (DAPI, blue) (middle row), and KIAA0319-GFP (lower row) for a representative RPE1 WT K-GFP cell (left column) and an Ex6KO K-GFP cell (right column). Yellow arrows point at accumulation of KIAA0319-GFP in the Golgi. Blue arrows point at KIAA0319-GFP in the plasma membrane. Scale bar, 20 µm.

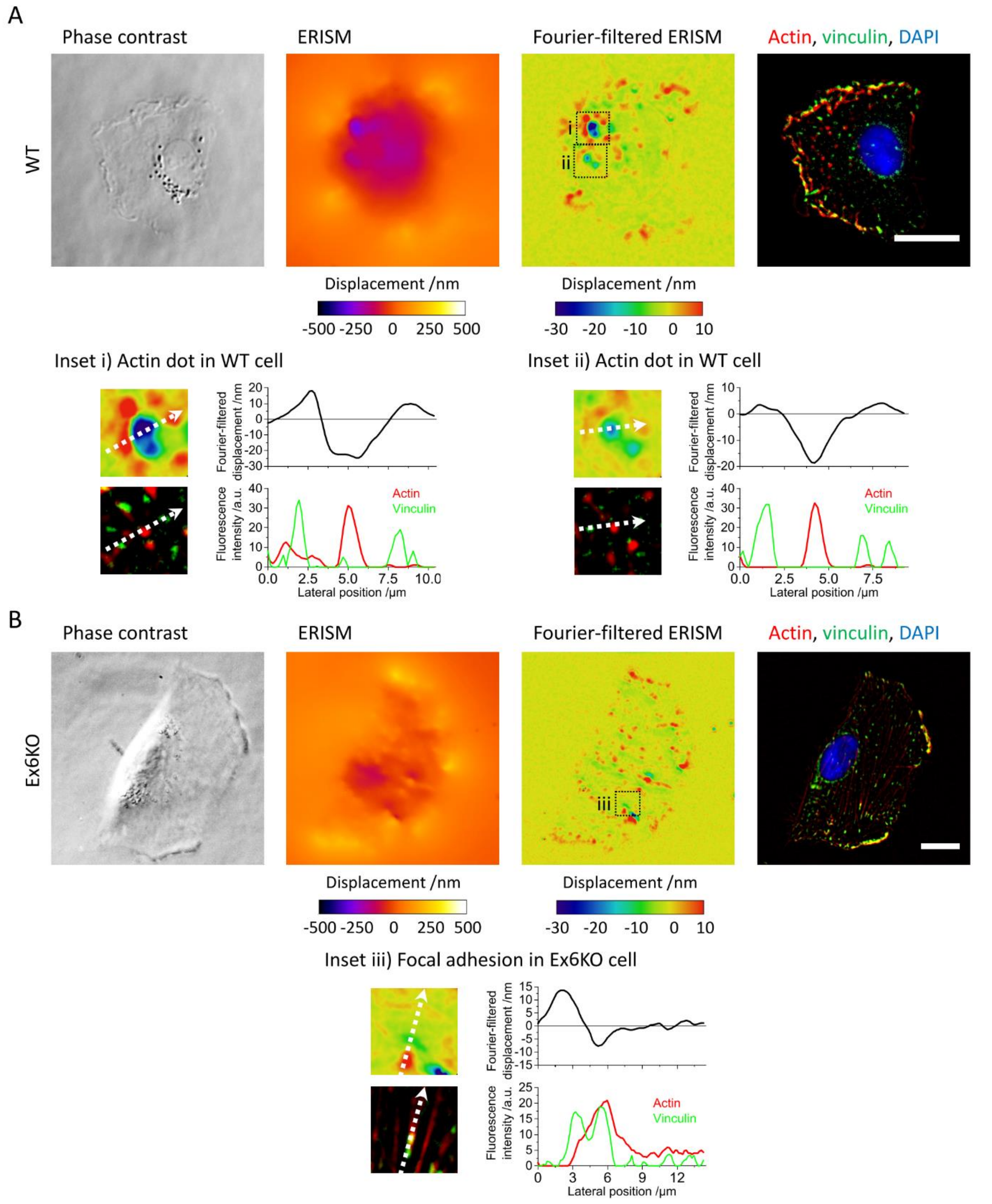

Figure S5. Fourier-filtered ERISM displacement map reveals two different types of mechanical cell-substrate interaction in RPE1 cells.

Phase contrast images (upper row, left), ERISM displacement map (upper row, middle left), Fourier-filtered ERISM displacement maps (upper row, middle right) and epi-fluorescence images (upper row, right; red: actin, green: vinculin, blue: nuclear DNA) of WT **(A)** and Ex6KO **(B)** cell. The lower rows in (A) and (B) show the areas marked i), ii) and iii) in the Fourier-filtered ERISM displacement maps

as magnified insets as well as the corresponding areas from the epi-fluorescence images, and topography and fluorescence intensity profiles measured along the dotted arrows in the insets. All scale bars: 20 µm.

Fourier-filtering of ERISM displacement maps reveals fine displacement features that are concealed in the unfiltered maps due to the overall cell contractility. Insets i) and ii) of the WT cell in (A) show tightly localised pushing sites in the Fourier-filtered ERISM map that colocalise with actin and are surrounded by rings of pulling sites that colocalise with vinculin. Inset iii) of the Ex6KO cell in (B) shows a push-pull pattern in the Fourier-filtered ERISM displacement map that colocalises with vinculin expression and is aligned along the direction of an actin stress fibre, suggesting that this feature is related to the torque exerted by a focal adhesion transmitting actomyosin contraction.

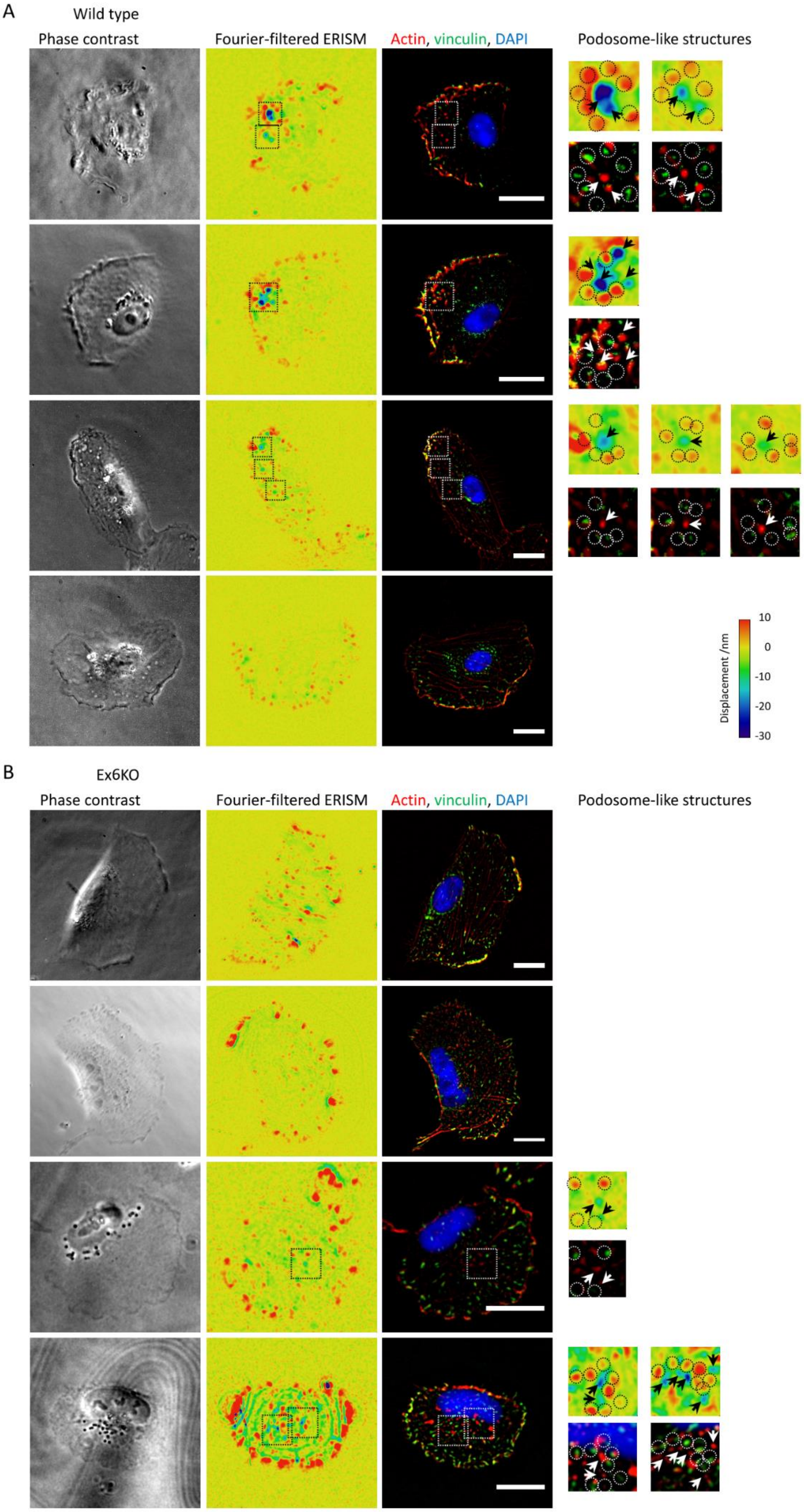

A
Wild type
Phase contrast
Fourier-filtered ERISM
Actin, vinculin, DAPI
Podosome-like structures
Displacement /nm
10
0
-10
-20
-30
B
Ex6KO
Phase contrast
Fourier-filtered ERISM
Actin, vinculin, DAPI
Podosome-like structures

Figure S6. RPE1 KIAA0319 WT and Ex6KO cells use different modes of force exertion.

Phase contrast images (left columns), Fourier-filtered ERISM displacement maps (centre columns) and epi-fluorescence images (right columns) of four fixed RPE1 WT cells **(A)** and four fixed Ex6KO cells **(B)** with staining for actin (red), vinculin (green) and nuclear DNA (blue). White arrows in the insets of the Fourier-filtered ERISM displacement maps and epi-fluorescence images indicate positions of actin-rich cell protrusions that are counterbalanced by pulling at vinculin-rich positions marked with circles. These structures are formed less often and less distinctly in Ex6KO cells as compared to WT cells. All scale bars: 20 µm.

Supplementary Videos

Movie S1. ERISM time-lapse investigation of mechanical activity of RPE1 WT cells

Time-lapse movie of phase contrast (left) and ERISM displacement (right) of RPE1 WT cells migrating on an ERISM substrate taken in intervals of five minutes over a time span of 17 hours.

Movie S2. ERISM time-lapse investigation of mechanical activity of RPE1 Ex6KO cells

Time-lapse movie of phase contrast (left) and ERISM displacement (right) of RPE1 Ex6KO cells migrating on an ERISM substrate taken in intervals of five minutes over a time span of 17 hours.

Movie S3. ERISM time-lapse investigation of mechanical activity of an RPE1 WT cell

Time-lapse movie of phase contrast (left) and Fourier-filtered ERISM displacement (right) of a RPE1 WT cell taken in intervals of five seconds over a time span of 12.5 minutes.

Movie S4. ERISM time-lapse investigation of mechanical activity of an RPE1 Ex6KO cell

Time-lapse movie of phase contrast (left) and Fourier-filtered ERISM displacement (right) of a RPE1 Ex6KO cell taken in intervals of five seconds over a time span of 12.5 minutes.

Movie S5. ERISM time-lapse investigation of mechanical activity of RPE1 WT cells shown in Figure S4.

Phase contrast image (left) and time-lapse movie of ERISM displacement (middle) and Fourier-filtered ERISM displacement (right) of three RPE1 WT cells taken in intervals of two minutes over a time span of 50 minutes. The displacement maps of the upper two cells show local, vertical force exertion by actin rich protrusions. Immunostainings of these two cells are shown in Figure S4.

Movie S6. ERISM time-lapse investigation of mechanical activity of RPE1 Ex6KO cells shown in Figure S5.

Phase contrast image (left) and time-lapse movie of ERISM displacement (middle) and Fourier-filtered ERISM displacement (right) of three RPE1 Ex6KO cells taken in intervals of two minutes over a time span of 42 minutes. The displacement maps of the lower two cells show local, vertical force exertion by actin rich protrusions. Immunostainings of these two cells are shown in Figure S4.

Supplementary Table

Table S1. Primer sequences

		**use**	**Size (in bp)**
int6-7R	ATCTAAGGTAATCTGCACTGGTGG	PCR	1311
int5-6F	AAATTAGCCGGGTGTGGTGAC		
ex11F	TCTTCAAGGCAACAGTCTACTG	qRT-PCR	128
ex12R	CCATCCAGGGTAGCACTTTC		
Ex6_R	AGAGTTTGCTTGTGTCCTTG	RT-PCR	137
Ex5_F	CCCGACAATGAAGTTGAACTG		
ex9R	ACGGGAGAGTCAACTGAAGTC	RT-PCR	360
ex6delF	CAACTATGAATGGAATTTAATAAGCCACC		
NHEJ gBR	AAACTGGTAGTCTGTGGGGTGGCTC	gRNA generation	
NHEJ gBF	CACCGAGCCACCCCACAGACTACCA		
NHEJ gAR	AAACACAACTATGAATGGAATTTAC		
NHEJ gAF	CACCGTAAATTCCATTCATAGTTGT		
