# Supplementary figures and images for: KIAA0319 influences cilia length, cell migration and mechanical cell–substrate interaction
Source: Sci Rep. 2022 Jan 14;12:722. doi: 10.1038/s41598-021-04539-3 (PMC8760330; doi:10.1038/s41598-021-04539-3)

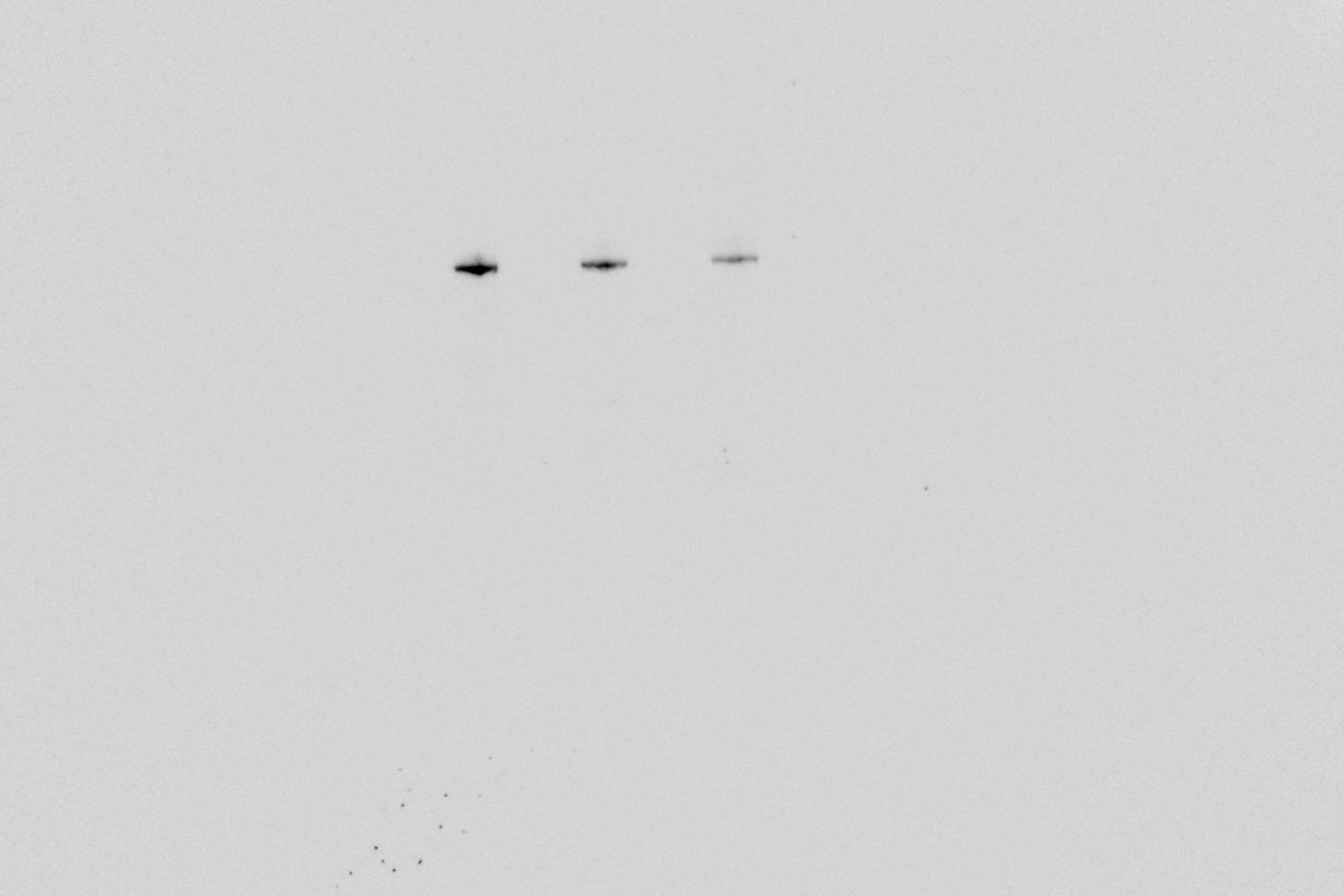

Supplement: Supplementary file 2 — Supplementary Information 2. [file 41598_2021_4539_MOESM2_ESM.tif]
